# Ropeginterferon alfa-2b every 2 weeks as a novel pegylated interferon for patients with chronic hepatitis B

**DOI:** 10.1007/s12072-020-10098-y

**Published:** 2020-10-24

**Authors:** Yi-Wen Huang, Chao-Wei Hsu, Sheng-Nan Lu, Ming-Lung Yu, Chien-Wei Su, Wei-Wen Su, Rong-Nan Chien, Ching-Sheng Hsu, Shih-Jer Hsu, Hsueh-Chou Lai, Albert Qin, Kuan-Chiao Tseng, Pei-Jer Chen

**Affiliations:** 1 Liver Center, Cathay General Hospital Medical Center, Taipei, Taiwan; 2 School of Medicine, College of Medicine, Taipei Medical University, Taipei, Taiwan; 3 School of Medicine, National Taiwan University College of Medicine, Taipei, Taiwan; 4 Division of Hepatogastroenterology, Department of Internal Medicine, Chang Gung Memorial Hospital, Chang Gung University College of Medicine, Taoyuan, Taiwan; 5 Division of Hepatogastroenterology, Department of Internal Medicine, Chia-Yi Chang Gung Memorial Hospital, Chia-Yi, Taiwan; 6 Hepatobiliary Section, Department of Internal Medicine and Hepatitis Center, Kaohsiung Medical University Hospital, Kaohsiung Medical University, Kaohsiung, Taiwan; 7 Division of Gastroenterology and Hepatology, Department of Internal Medicine, Taipei Veterans General Hospital, Taipei, Taiwan; 8 Division of Gastroenterology and Hepatology, Department of Internal Medicine, Changhua Christian Hospital, Changhua, Taiwan; 9 Division of Hepatogastroenterology, Department of Internal Medicine, Keelung Chang Gung Memorial Hospital, Keelung, Taiwan; 10 Liver Diseases Research Center, Taipei Tzu Chi Hospital, Buddhist Tzu Chi Medical Foundation, Taipei, Taiwan; 11 Division of Gastroenterology and Hepatology, Department of Internal Medicine, National Taiwan University Hospital Yun-Lin Branch, Yunlin, Taiwan; 12 Division of Hepatogastroenterology, Department of Internal Medicine, China Medical University Hospital, China Medical University, Taichung, Taiwan; 13 PharmaEssentia Corp, Taipei, Taiwan; 14 Graduate Institute of Clinical Medicine, National Taiwan University College of Medicine, No. 7, Chung-Shan South Rd., Taipei, Taiwan; 15 Hepatitis Research Center, National Taiwan University Hospital, Taipei, Taiwan

**Keywords:** Clinical trial, Pegylated interferon, P1101, Besremi, Hepatitis B virus, HBeAg seroconversion, Antiviral, Infectious disease, Therapy, Taiwan

## Abstract

**Background:**

Ropeginterferon alfa-2b is a novel mono-pegylated interferon that has only one major form as opposed to 8–14 isomers of other on-market pegylated interferon, allowing injection every two or more weeks with higher tolerability. It received European Medicines Agency and Taiwan marketing authorization in 2019 and 2020, for treatment of polycythemia vera. This phase I/II study aimed to have preliminary evaluation of safety and efficacy in chronic hepatitis B.

**Methods:**

Thirty-one HBeAg-positive and 31 HBeAg-negative were stratified by HBeAg status and randomized at 1:1:1 ratio to q2w ropeginterferon alfa-2b 350 μg (group 1), q2w 450 μg (group 2) or q1w PEG-IFN alfa-2a 180 μg (group 3). Each patient received 48-week treatment (TW48) and 24-week post-treatment follow-up (FW24).

**Results:**

The baseline demographics were comparable among the three groups, except for mean HBeAg in HBeAg-positive patients (2.90, 2.23, 2.99 log_10_ S/CO, respectively). Cumulative HBeAg seroconversion rate at follow-up period was 27.3% (3/11), 36.4% (4/11), and 11.1% (1/9) with time to HBeAg seroconversion starting from TW24, TW16, and TW48 in group 1, 2, and 3, respectively. The rate of HBV DNA < 2000 IU/mL and HBsAg levels < 1500 IU/mL at FW24 were comparable in all groups. Ropeginterferon alfa-2b (group 1 & 2) had numerically lower incidence of rash (9.5% and 4.5%) as compared to PEG-IFN alfa-2a (36.8%). Ropeginterferon alfa-2b 350 μg (group 1) had more ALT elevation (38.1%), however the rate was comparable in group 2 (9.1%) and group 3 (10.5%).

**Conclusion:**

In this preliminary study, ropeginterferon alfa-2b, although in only half the number of injections, is as safe and effective as pegylated interferon alfa-2a for chronic hepatitis B.

**Graphic abstract:**

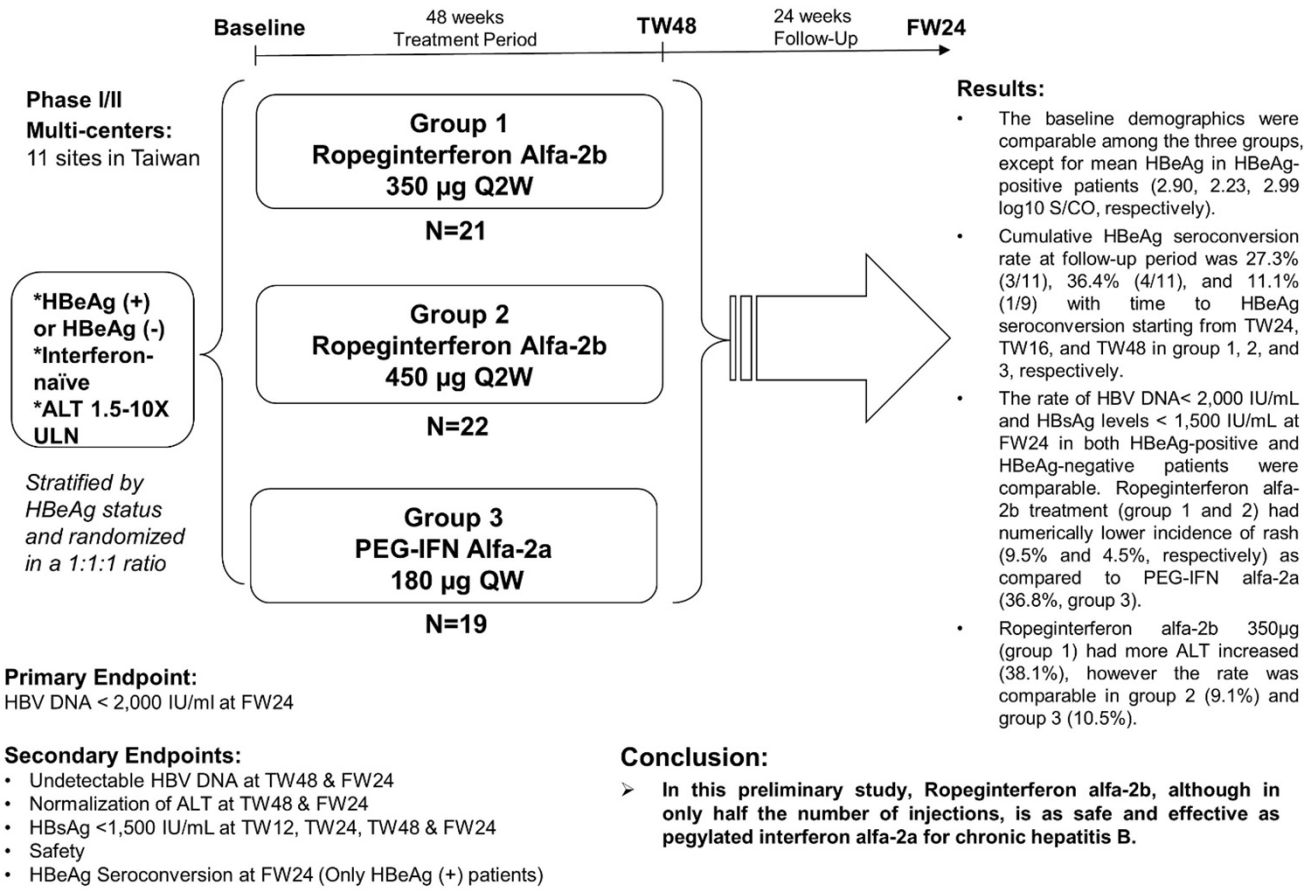

**Electronic supplementary material:**

The online version of this article (10.1007/s12072-020-10098-y) contains supplementary material, which is available to authorized users.

## Introduction

Chronic hepatitis B virus (HBV) infection is a major public health problem worldwide and affects over 257 million people globally [1]. Pegylated interferon (PEG-IFN) alfa and nucleos(t)ide analogues (NUCs) are approved for treatment of chronic HBV infection. PEG-IFN alfa, entecavir and tenofovir are the preferred first-line treatment for chronic HBV infection.

NUCs lead to on-treatment suppression of HBV DNA, regression of hepatic fibrosis and cirrhosis, reduction of disease progression and hepatocellular carcinoma [2, 3]. However, HBsAg clearance is difficult to achieve by NUCs as HBV is integrated in the host genome and persists in covalently closed circular DNA form in the hepatocyte, even after HBV DNA is not detectable in the serum [4, 5]. NUCs have the advantage as oral form, but their effect on virus elimination or off-treatment permanent suppression is limited.

PEG-IFN alfa therapy leads to higher rates of HBeAg and HBsAg seroconversion than NUCs. HBeAg seroconversion and HBsAg clearance represent an immune control of the chronic HBV infection. PEG-IFN alfa treatment of 48 weeks’ duration yields HBeAg seroconversion rates of 20–31%. These endpoints of sustained off-therapy virological response and HBeAg seroconversion are more desirable than only maintained virological remission under continuous NUCs. Sustained HBeAg and HBsAg negativity up to 37% and 11%, respectively, were observed in HBeAg-positive patients treated with PEG-IFN alfa at a mean follow-up of 3 years. Among those with HBeAg loss at 26 weeks post-treatment, sustained HBeAg and HBsAg negativity were up to 81% and 30% [6]. Although PEG-IFN alfa has the advantages of sustained off-therapy response, premature discontinuation had been reported in up to 20% during chronic hepatitis B treatment [7]. Among the side effects of PEG-IFN, depression and suicidal ideation is the one that correlates with poorer treatment response independent of dose reduction and needs special precaution [8]. Previously, Novartis developed albinterferon dosed every two weeks for treatment of chronic viral hepatitis, aiming to reduce injection frequency and adverse events of weekly PEG-IFN. However, the project had to be discontinued after lung toxicities were noted [9–11].

Mono-PEGylated interferon alfa-2b (INN: ropeginterferon alfa-2b; P1101; Besremi™) has been developed by site-specific conjugation of a 40 kDa branched mPEG polymer to an engineered proline-IFN alfa-2b molecule. The generation of pro-IFN alfa-2b, recombinantly expressed in bacteria by engineering an extra proline at the N-terminus of human IFN alfa-2b, and the redesigned PEG moiety allow specific PEGylation to preferentially occur at the N-terminal proline. Thus, ropeginterferon alfa-2b is a novel mono-pegylated IFN alfa-2b with unique structural characteristics that has only one major form as opposed to the 8–14 isomers of other PEGylated IFN alfa products (US Patent No. 8143214. 2012-03-27.) (Counterpart: TW Patent No. I381851. 2013-01-11; JP Patent No. 5613050. 2014-09-12; KR Patent No. 10/15888465. 2016-01-19; AU Patent No. 2008286742. 2014-09-11; CA Patent No. 2696478. 2018-10-09).

Ropeginterferon alfa-2b has longer duration of action, allowing injection every two or more weeks with higher tolerability. Ropeginterferon alfa-2b received European Medicines Agency marketing authorization in 2019 (https://www.ema.europa.eu/en/medicines/human/EPAR/besremi) and Taiwan marketing authorization in 2020 for the treatment of polycythemia vera. In contrast, weekly PEG-IFN alfa-2a had been studied in PV patients but was eventually given up by hematologists because of intolerance [12, 13] and is not approved for PV. This shows a clear advantage of biweekly ropeginterferon alfa-2b over weekly PEG-IFN alfa-2a in the PV patient population in terms of compliance/tolerance, which potentially can be extended to hepatitis B patients.

In phase I study of ropeginterferon alfa-2b, a total of 48 healthy subjects were enrolled. There were 6 cohorts and each cohort enrolled 6 subjects who received a single dose of ropeginterferon alfa-2b (24, 48, 90, 180, 225, 270 μg) and 2 subjects who received a single dose of PEG-IFN alfa-2a (180 μg). In safety part, majority of reported AEs were mild or moderate in intensity. No clinically meaningful changes were observed in laboratory assessments, physical examinations, vital signs measurements, or ECG results. In pharmacokinetics (PK) results, the geometric mean values of ropeginterferon alfa-2b for *C*_max_, AUC and AUC_0-t_ showed an increase of 76%, 66%, and 82%, respectively compared to PEG-IFN alfa-2a at the 180 μg dose level. Mean Pharmacodynamics (PD) parameters (*E*_max_, *T*_max_, and AUC_0-t_) of ropeginterferon alfa-2b increased with dose for both neopterin and 2, 5- OAS. The PD data showing no statistically significant difference between ropeginterferon alfa-2b and PEG-IFN alfa-2a at the 180 μg dose level. (unpublished data). Furthermore, ropeginterferon alfa-2b dose up to 450 µg was safe and well-tolerated in patients with chronic hepatitis C virus genotype 1 and 2 infection, both studies were completed [14, 15]. In this study, we aimed to have preliminary evaluation of safety and efficacy of ropeginterferon alfa-2b in chronic HBV-infected patients.

## Methods

### Study population

Adult patients with the following criteria at screening were eligible: positive HBsAg ≧ 6 months; serum ALT levels 1.5–10 times ULN; serum HBV DNA levels > 20,000 IU/mL for HBeAg-positive and > 2000 IU/mL for HBeAg-negative patients; compensated liver disease with total bilirubin < 2 mg/dL, albumin levels ≧ 3.5 g/dL, INR ≦ 1.5; interferon treatment naïve; no other form of chronic liver disease; no significant steatohepatitis on ultrasound or other procedures; negative for human immunodeficiency virus, hepatitis C and hepatitis D infection; normal fundoscopic examination at screening.

Patients with any of the following criteria at screening were excluded: clinically significant vital sign abnormalities, hemoglobin < 10 g/dL, WBC count < 3000/mm^3^, absolute neutrophil count < 1500/mm^3^, platelet count < 90,000/mm^3^, abnormal serum creatinine, alcohol or substance abuse, poorly controlled psychiatric or systemic disease, coagulation disorders, a depot injection or an implant of any drug within 3 months prior to administration of study medication other than contraception, organ transplant and are taking immunosuppressants, cancers (except those considered cured), history of opportunistic infection, serious localized or systemic infection within 3 months prior to screening, any other clinical conditions that may interfere with study participation or absorption, distribution, metabolism or excretion of the study drug, pregnancy or unwillingness to abstain from contraception. Any systemic antiviral treatment, including nucleos(t)ide analogues for HBV, anti-neoplastic, and immunomodulatory agents should be washed out for at least 1 month or 3 months for those with longer elimination half-lives prior to the first dose of the study drug.

### Study design

**Fig. 1:**
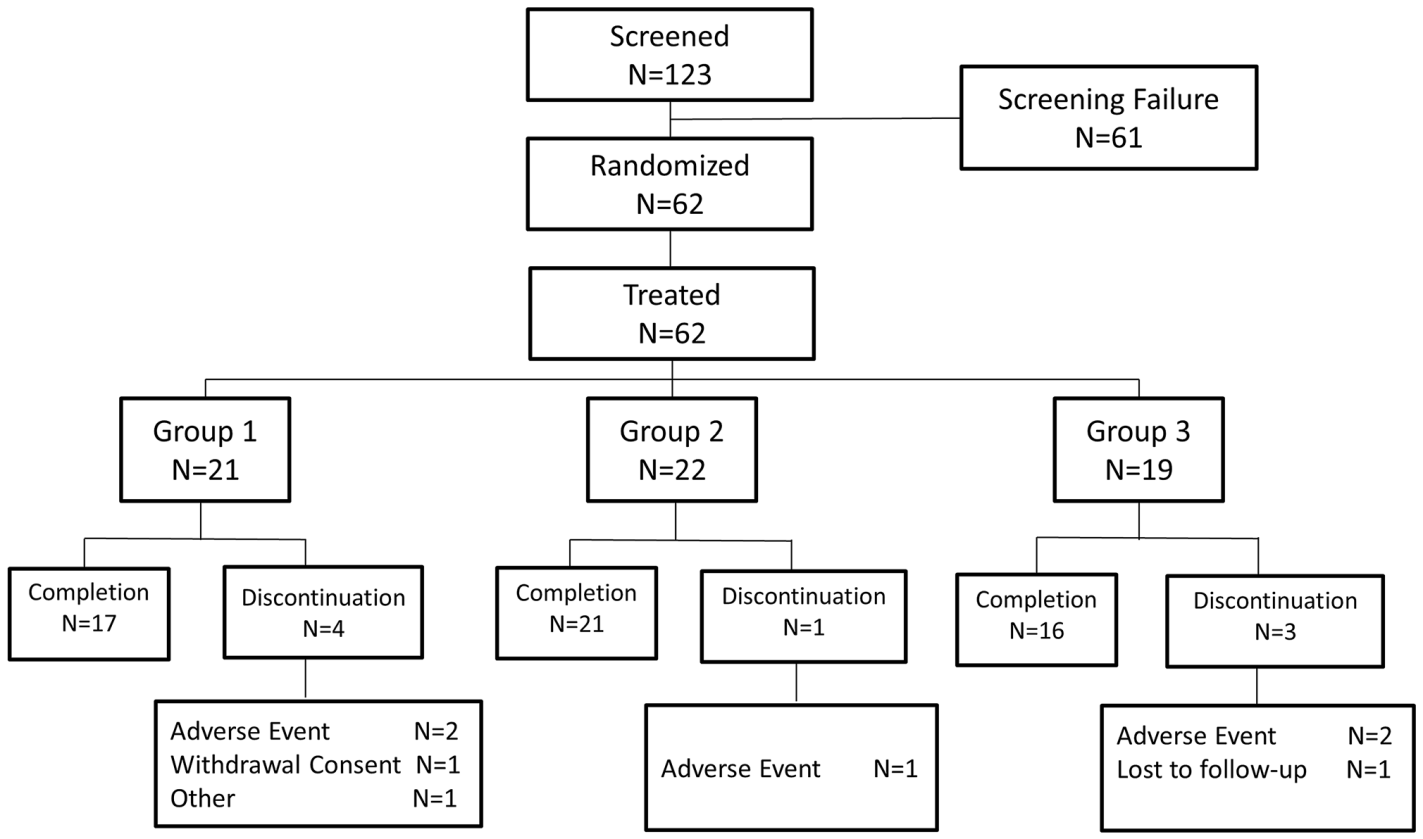
Flow chart of patient enrollment. Group 1, Ropeginterferon alfa-2b 350 μg every 2 weeks (q2w); Group 2, Ropeginterferon alfa-2b 450 μg every 2 weeks (q2w); group 3, PEG-IFN alfa-2a 180 μg weekly (qw)

This was a Phase I/II multicenter, open-label, randomized, active control, dose finding study conducted at 11 medical centers in Taiwan between 2014 and 2017. Total of 62 patients were enrolled, 31 hepatitis B e-antigen (HBeAg)-positive patients and 31 HBeAg-negative patients were stratified by HBeAg status and randomized at 1:1:1 ratio to ropeginterferon alfa-2b 350 μg q2w (group 1), ropeginterferon alfa-2b 450 μg q2w (group 2) or PEG-IFN alfa-2a 180 μg qw (group 3) (Fig. 1). Every patient received 48-week treatment (i.e. 24 injections per patient in ropeginterferon alfa-2b groups and 48 injections per patient in the PEG-IFN alfa-2a group) and 24 weeks post-treatment follow-up. The study was completed on Sep 2, 2017.

Ropeginterferon alfa-2b was supplied in a sterile, single-use prefilled syringe (500 μg/mL/syringe; PharmaEssentia Corp., Taiwan). PEG-IFN alfa-2a was supplied in a sterile, single-use prefilled syringe (PEGASYS^®^, 180 μg/0.5 ml/ syringe; F. Hoffmann-La Roche Ltd, Basel, Switzerland). Ropeginterferon alfa-2b was injected subcutaneously once every 2 weeks, while the active comparator was injected subcutaneously once every week.

### Assessment and endpoints

The primary efficacy endpoint was HBV DNA < 2000 IU/mL at 24 weeks of follow-up. Secondary endpoints include the rate of HBV DNA < 2000 IU/mL, and ALT normalization (≦ ULN) at the end of 48 weeks treatment and at 24 weeks of follow-up; HBsAg < 1500 IU/mL at 12 weeks, 24 weeks, 48 weeks of treatment, and at 24 weeks of follow-up; HBeAg seroconversion, defined as loss of HBeAg and the development of anti-HBe in HBeAg-positive patients; and safety throughout the study. The analyses of efficacy endpoints were performed in ITT and PP population. The conclusion was based on the results of ITT population.

Serum HBV DNA levels were measured by TaqMan HBV Test (Roche, lower limit of detection, [LOD], < 20 IU/mL). Serum HBsAg levels (LOD < 0.05 IU/mL) and HBeAg levels (LOD: < 1.0 signal/cut-off, S/CO) were measured by Chemiluminescent Microparticle Immunoassay (Abbott ARCHITECT^®^ HBsAg, HBeAg reagent kit). The ALT value was tested at the study site laboratory and the upper limit of normal at each study site ranged from 30 to 41 U/L. The assessment of AE was based on the investigator’s clinical judgment with reference to MedDRA coding and NCI-CTCAE version 4.0.

### Statistical analyses

Efficacy analyses were performed using intent-to-treat (ITT) population and per-protocol (PP) population. ITT population included all randomized patients receiving ≥ 1 dose of study drug and ≥ 1 evaluable follow-up evaluation. PP population included all randomized patients receiving at least 80% compliance of study drug, ≥ 1 evaluable follow-up evaluation, and completed the study without major protocol violations. The safety population included all patients receiving ≥ 1 dose of study drug. Statistical analyses were conducted using SAS version 9.4 or higher.

### Sample size

The sample size of this study was not statistically powered and the analyses were not hypotheses driven but exploratory in nature. We expect to have the preliminary evaluation of safety and efficacy across the treatment groups by 20 patients in each group. The results will be used to plan future hepatitis B trials.

## Results

### Patient characteristics

In this study, a total of 123 subjects were screened; 61 (49.6%) subjects were not randomized due to failure to fulfill the inclusion and exclusion criteria or withdrawal consent. Of these 61 subjects with screening failure (Supplemental Table 1), 51 subjects did not meet inclusion criteria #2 (ALT elevation or HBV DNA level did not meet). Additional 2, 1 and 2 of them did not meet the inclusion criteria #3, #6 and #7, respectively. Furthermore, 2 subjects were excluded by exclusion criteria #3 and 1 subject was excluded by exclusion criteria #10. The other two subjects withdrew during the screening period.

**Table 1:** Demographic data and baseline characteristics (ITT population)

	P1101/350 μg (*N* = 21)	P1101/450 μg (*N* = 22)	PEG-IFN alfa-2a 180 μg (*N* = 19)
Age, years			
Median	43.4	41.4	43.6
Range (Min–Max)	23.7–62.0	30.5–67.9	30.9–73.8
Male, *n* (%)	16 (76.2)	16 (72.7)	14 (73.7)
Race, *n* (%)			
Asian	21 (100.0)	22 (100.0)	19 (100.0)
HBeAg, *n* (%)			
Positive	11 (52.4)	11 (50.0)	9 (47.4)
ALT level (U/L)			
Median	99.0	142.0	115.0
Range (Min–Max)	57.0–227.0	57.0–358.0	56.0–403.0
ALT × ULN, *n* (%)			
> 1.5 to ≤ 2× ULN	2 (9.5)	2 (9.1)	4 (21.1)
> 2 to ≤ 5× ULN	18 (85.7)	16 (72.7)	12 (63.2)
> 5 to ≤ 10× ULN	1 (4.8)	4 (18.2)	3 (15.8)

HBeAg positive	P1101/350 μg (*N* = 11)	P1101/450 μg (*N* = 11)	PEG-IFN alfa-2a 180ug (*N* = 9)
HBV DNA (Log_10_ IU/mL) Median			
	8.0	7.9	8.1
Range (Min–Max)	7.6–8.6	6.4–9.0	6.9–8.9
HBsAg (Log_10_ IU/mL)			
Median	4.2	4.0	4.2
Range (Min–Max)	3.3–4.8	2.9–4.9	3.2–5.1
HBeAg (Log_10_ S/CO)			
Median	3.04	2.42	3.03
Range (Min–Max)	2.10–3.22	0.02–3.26	2.60–3.18
ALT (U/L) at TW0			
Median	132.0	137.0	130.0
Range (Min–Max)	61.0–426.0	33.0–385.0	41.0–307.0

HBeAg negative	P1101/350 μg (*N* = 10)	P1101/450 μg (*N* = 11)	PEG-IFN alfa-2a 180 μg (*N* = 10)
HBV DNA (Log_10_ IU/mL)			
Median	5.6	5.6	5.4
Range (Min–Max)	4.6–6.3	3.2–8.2	3.7–7.0
HBsAg (Log_10_ IU/mL)			
Median	2.9	3.2	2.7
Range (Min–Max)	1.6–3.6	1.8–3.4	1.2–3.3
ALT (U/L) at TW0			
Median	77.0	94.0	72.0
Range (Min–Max)	32.0–323.0	42.0–201.0	33.0–283.0

*P1101* Ropeginterferon alfa-2b, *NA* not applicable;

Sixty-two patients were enrolled to the study (Fig. 1) and were randomized into 3 treatment groups: 21 in group 1, 22 in group 2, and 19 in group 3. The mean age, gender, race, HBeAg status, ALT level were comparable among three groups (Table 1). Baseline HBV DNA level, HBsAg level, and ALT level were comparable in both HBeAg-positive and HBeAg-negative patients. In HBeAg-positive, patients in group 2 had lower baseline mean HBeAg level, 2.23 log_10_ S/CO as compared to either group 1 (2.90 log_10_ S/CO) or group 2 (2.99 log_10_ S/CO).

### Safety

A total of 598 treatment emergent adverse events (TEAEs) were reported. Among them, 432 TEAEs in 57 patients were reported as related to the study drug (i.e. related TEAEs in Table 2). One (4.8%) SAE in group 1, the patient was hospitalized due to myocardial infarction. No death was reported in this study. Related TEAEs leading to dose discontinuation were comparable among the three groups.

**Table 2:** Safety summary (safety population)

	Severity (grade)	P1101/350 μg (*N* = 21)	P1101/450 μg (*N* = 22)	PEG-IFN alfa-2a 180 μg (*N* = 19)
Any TEAEs		183, 21 (100)	245, 22 (100)	170, 19 (100)
Related TEAEs	Total	135, 18 (85.7)	171, 22 (100)	126, 17 (89.5)
	1	108, 7 (33.3)	145, 8 (36.4)	110, 6 (31.6)
	2	13, 1 (4.8)	19, 9 (40.9)	11, 7 (36.8)
	3	11, 8 (38.1)	7, 5 (22.7)	4, 3 (15.8)
	4	3, 2 (9.5)	0, 0 (0)	1, 1 (5.3)
SAE		1, 1 (4.8)	0, 0 (0)	0, 0 (0)
TEAEs leading to dose discontinuation		2**,** 2 (9.5)	8**,** 1 (4.5)	2**,** 2 (10.5)
Related TEAEs leading to dose discontinuation		2**,** 2 (9.5)	7**,** 1 (4.5)	1**,** 1 (5.3)
Related TEAE ≥ 10% in any group				
Leukopenia	Total	5, 2 (9.5)	27, 9 (40.9)	10, 5 (26.3)
	1	5, 2 (9.5)	27, 9 (40.9)	9, 4 (21.1)
	2	0, 0 (0)	0, 0 (0)	1, 1 (5.3)
Neutropenia	Total	20, 10 (47.6)	38, 18 (81.8)	21, 11 (57.9)
	1	17, 7 (33.3)	28, 11 (50.0)	16, 6 (31.6)
	2	1, 1 (4.8)	7, 4 (18.2)	3, 3 (15.8)
	3	2, 2 (9.5)	3, 3 (13.6)	1, 1 (5.3)
	4	0, 0 (0)	0, 0 (0)	1, 1 (5.3)
Thrombocytopenia	Total	16, 10 (47.6)	14, 8 (36.4)	7, 4 (21.1)
	1	16, 10 (47.6)	11, 5 (22.7)	6, 3 (15.8)
	2	0, 0 (0)	3, 3 (13.6)	1, 1 (5.3)
Nausea	Total	0, 0 (0)	0, 0 (0)	2, 2 (10.5)
	1	0, 0 (0)	0, 0 (0)	2, 2 (10.5)
Fatigue	Total	1, 1 (4.8)	1, 1 (4.5)	7, 5 (26.3)
	1	1, 1 (4.8)	1, 1 (4.5)	7, 5 (26.3)
Pyrexia	Total	2, 2 (9.5)	4, 3 (13.6)	3, 2 (10.5)
	1	2, 2 (9.5)	4, 3 (13.6)	3, 2 (10.5)
Upper respiratory tract infection	Total	2, 2 (9.5)	5, 3 (13.6)	0, 0 (0)
	1	1, 1 (4.8)	2, 2 (9.1)	0, 0 (0)
	2	1, 1 (4.8)	3, 1 (4.5)	0, 0 (0)
Alanine Aminotransferase increased	Total	8, 8 (38.1)	2, 2 (9.1)	2, 2 (10.5)
	2	1, 1 (4.8)	0, 0 (0)	0, 0 (0)
	3	5, 5 (23.8)	2, 2 (9.1)	2, 2 (10.5)
	4	2, 2 (9.5)	0, 0 (0)	0, 0 (0)
Aspartate Aminotransferase increased	Total	6, 6 (28.6)	1, 1 (4.5)	2, 2 (10.5)
	2	2, 2 (9.5)	0, 0 (0)	1, 1 (5.3)
	3	3, 3 (14.3)	1, 1 (4.5)	1, 1 (5.3)
	4	1, 1 (4.8)	0, 0 (0)	0, 0 (0)
Weight decreased	Total	5, 5 (23.8)	3, 2 (9.1)	4, 3 (15.8)
	1	5, 5 (23.8)	2, 1 (4.5)	4, 3 (15.8)
	2	0, 0 (0)	1, 1 (4.5)	0, 0 (0)
White blood cell count decreased	Total	12, 5 (23.8)	6, 5 (22.7)	3, 2 (10.5)
	1	12, 5 (23.8)	5, 4 (18.2)	3, 2 (10.5)
	3	0, 0 (0)	1, 1 (4.5)	0, 0 (0)
Myalgia	Total	3, 2 (9.5)	7, 7 (31.8)	3, 3 (15.8)
	1	2, 1 (4.8)	7, 7 (31.8)	3, 3 (15.8)
	2	1, 1 (4.8)	0, 0 (0)	0, 0 (0)
Dizziness	Total	1, 1 (4.8)	1, 1 (4.5)	2, 2 (10.5)
	1	1, 1 (4.8)	1, 1 (4.5)	2, 2 (10.5)
Headache	Total	9, 2 (9.5)	7, 6 (27.3)	6, 5 (26.3)
	1	9, 2 (9.5)	5, 4 (18.2)	5, 4 (21.1)
	2	0, 0 (0)	2, 2 (9.1)	1, 1 (5.3)
Insomnia	Total	0, 0 (0)	4, 4 (18.2)	3, 2 (10.5)
	1	0, 0 (0)	4, 4 (18.2)	3, 2 (10.5)
Cough	Total	2, 2 (9.5)	2, 2 (9.1)	2, 2 (10.5)
	1	2, 2 (9.5)	2, 2 (9.1)	2, 2 (10.5)
Alopecia	Total	5, 5 (23.8)	6, 6 (27.3)	3, 3 (15.8)
	1	5, 5 (23.8)	6, 6 (27.3)	3, 3 (15.8)
Pruritus	Total	2, 2 (9.5)	5, 5 (22.7)	6, 5 (26.3)
	1	1, 1 (4.8)	5, 5 (22.7)	6, 5 (26.3)
	2	1, 1 (4.8)	0, 0 (0)	0, 0 (0)
Rash	Total	2, 2 (9.5)	1, 1 (4.5)	8, 7 (36.8)
	1	1, 1 (4.8)	1, 1 (4.5)	8, 7 (36.8)
	2	1, 1 (4.8)	0, 0 (0)	0, 0 (0)
Diarrhea	Total	1, 1 (4.8)	0, 0 (0)	3, 3 (15.8)
	1	1, 1 (4.8)	0, 0 (0)	3, 3 (15.8)
Dry Mouth	Total	0, 0 (0)	0, 0 (0)	2, 2 (10.5)
	1	0, 0 (0)	0, 0 (0)	2, 2 (10.5)
Malaise	Total	0, 0 (0)	2, 2 (9.1)	2, 2 (10.5)
	1	0, 0 (0)	2, 2 (9.1)	1, 1 (5.3)
	2	0, 0 (0)	0, 0 (0)	1, 1 (5.3)

Data presented by number of events, number of patients (% of patients) with TEAEs

*P1101* Ropeginterferon alfa-2b, *TEAE* treatment emergent adverse event, *related TEAE*= TEAE related to the study drug, *NA* not applicable

Related TEAEs of ≧10% were shown in Table 2. No depression was reported in this study. Ropeginterferon alfa-2b treatment (group 1 and 2) had numerically lower incidence of rash (9.5% and 4.5%, respectively) as compared to PEG-IFN alfa-2a (36.8%, group 3). The rate of ALT elevation was higher in group 1 (38.1%), but not in group 2 (9.1%) or group 3 (10.5%). Additional analysis on the rate of subsequent HBV DNA suppression after the ALT elevation had no huge difference among the three groups (60%, 75%, 50%, respectively). Interestingly, the rate of subsequent HBeAg seroconversion after the ALT elevation was 20% and 25% in group 1 and 2 (ropeginterferon alfa-2b arm) but not seen in PEG-IFN alfa-2a arm.

### Efficacy

The efficacy data were shown in Table 3 (ITT population). Data of PP population were shown in Supplemental Table 2. In HBeAg-positive and HBeAg-negative patients, the rate of HBV DNA < 2000 IU/mL during treatment and follow-up among the three groups was similar. In HBeAg-positive and HBeAg-negative patients, the rate of HBsAg levels < 1500 IU/mL during treatment and follow-up among the three groups was also similar.

**Table 3:** Efficacy summary (ITT population)

	HBeAg (+)			HBeAg (−)		
	P1101/350 μg (*N* = 11)	P1101/450 μg (*N* = 11)	PEG-IFN alfa-2a 180 μg (*N* = 9)	P1101/350 μg (*N* = 10)	P1101/450 μg (*N* = 11)	PEG-IFN alfa-2a 180 μg (*N* = 10)
HBV DNA level < 2000 IU/mL						
TW4	0 (0.0)	0 (0.0)	0 (0.0)	8 (80.0)	6 (54.5)	6 (60.0)
TW8	1 (9.1)	0 (0.0)	0 (0.0)	9 (90.0)	7 (63.6)	9 (90.0)
TW12	1 (9.1)	1 (9.1)	1 (11.1)	10 (100.0)	9 (81.8)	9 (90.0)
TW24	2 (18.2)	1 (9.1)	1 (11.1)	9 (90.0)	9 (81.8)	9 (90.0)
TW48	3 (27.3)	4 (36.4)	2 (22.2)	9 (90.0)	10 (90.9)	9 (90.0)
FW12	2 (18.2)	1 (9.1)	1 (11.1)	3 (30.0)	6 (54.5)	7 (70.0)
FW24	2 (18.2)	0 (0.0)	2 (22.2)	1 (10.0)	4 (36.4)	5 (50.0)
HBsAg levels < 1500 IU/mL						
TW12	1 (9.1)	2 (18.2)	1 (11.1)	5 (50.0)	7 (63.6)	9 (90.0)
TW24	2 (18.2)	2 (18.2)	2 (22.2)	6 (60.0)	8 (72.7)	9 (90.0)
TW48	4 (36.4)	3 (27.3)	3 (33.3)	9 (90.0)	9 (81.8)	10 (100.0)
FW24	4 (36.4)	2 (18.2)	1 (11.1)	8 (80.0)	10 (90.9)	9 (90.0)
HBeAg seroconversion						
TW24	2 (18.2)	3 (27.3)	0 (0.0)	NA	NA	NA
TW48	2 (18.2)	4 (36.4)	1 (11.1)	NA	NA	NA
FW12	3 (27.3)	4 (36.4)	1 (11.1)	NA	NA	NA
FW24	2^a^ (18.2)	4 (36.4)	1 (11.1)	NA	NA	NA

*NA* not applicable, *P1101* ropeginterferon alfa-2b

^a^One patient was treated with Telbivudine after FW12 and withdrawn from study

With regard of HBsAg reduction over time, in HBeAg-positive patient, the rate of reduction > 1 log_10_ at FW24 was 0% (0/11), 0% (0/11), and 11.1% (1/9) in group 1, 2, and 3, respectively. In HBeAg-negative patients at FW24, the rate was 10.0% (1/10), 45.5% (5/11), and 30.0% (3/10), respectively.

In HBeAg-positive and HBeAg-negative patients, the rate of ALT normalization during treatment and follow-up among the three groups was similar.

### Trend of HBeAg seroconversion in HBeAg-positive patients treated with ropeginterferon alfa-2b

**Fig. 2:**
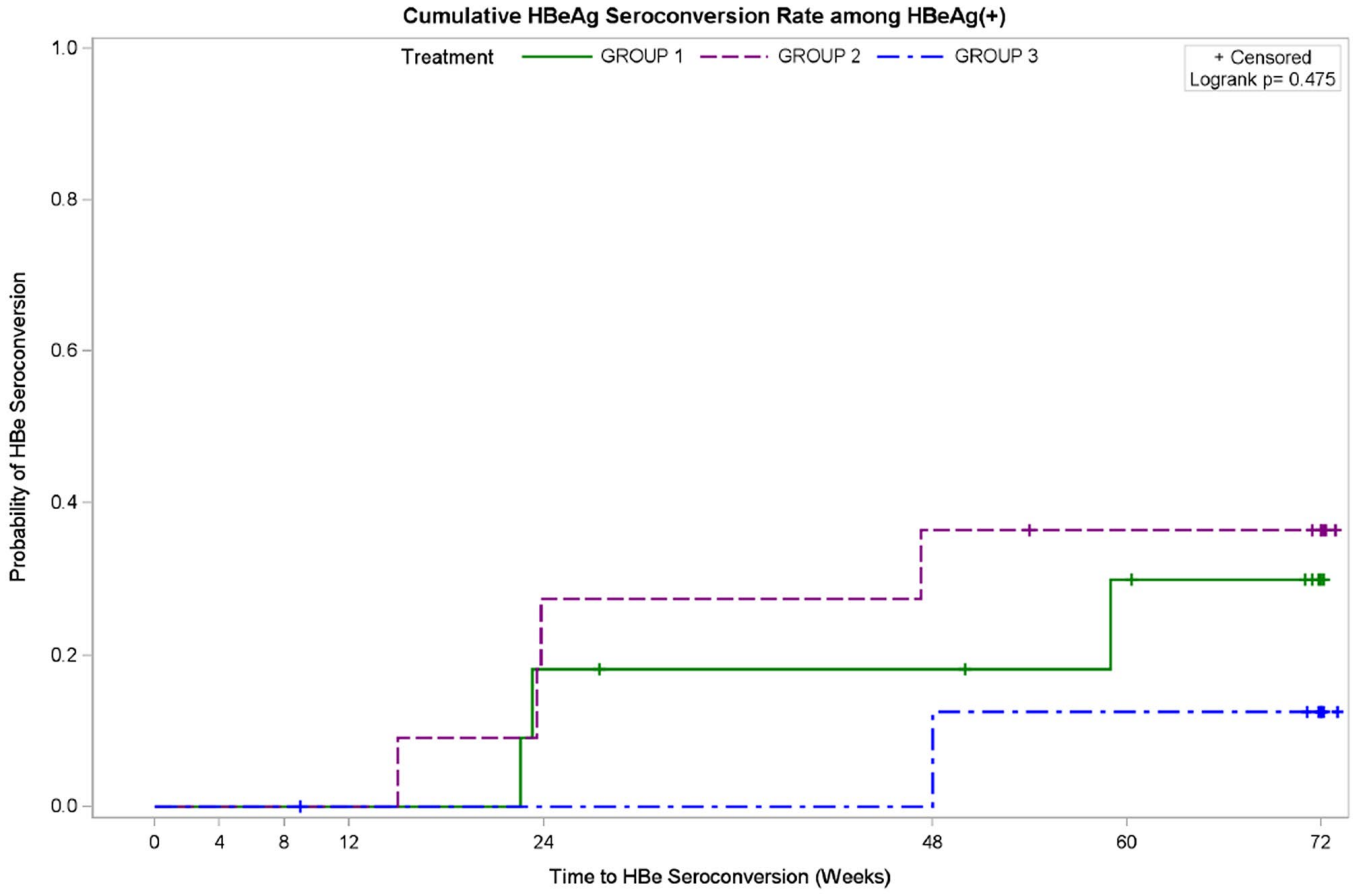
The cumulative rate and time to HBeAg seroconversion among HBeAg-positive patients within the three treatment groups. Group 1, Ropeginterferon alfa-2b 350 μg every 2 weeks (q2w); group 2, Ropeginterferon alfa-2b 450 μg every 2 weeks (q2w); group 3, PEG-IFN alfa-2a 180 μg weekly (qw)

Patients in ropeginterferon alfa-2b groups (both group 1 and 2) experienced earlier time to HBeAg seroconversion than PEG-IFN alfa-2a group (group 3) (Fig. 2). Patients treated with ropeginterferon alfa-2b 450 μg (group 2) experienced HBeAg seroconversion starting from treatment week (TW) 16 and those treated with ropeginterferon alfa-2b 350 μg (group 1) developed HBeAg seroconversion starting from TW24. In contrast, patients treated with PEG-IFN alfa-2a 180 μg (group 3) experienced HBeAg seroconversion starting from TW48.

At follow-up week 12, the rate of HBeAg seroconversion was 27.3% (3/11) and 36.4% (4/11) in group 1 and group 2, respectively, as compared to 11.1% (1/9) in group 3. One patient in group 1 was treated with Telbivudine after FW12 and withdrawn from study (Table 3).

Table 4 showed the analysis for predictors of HBeAg seroconversion (ITT population). Data of PP population were shown in Supplemental Table 3. Univariate analysis revealed lower baseline HBV DNA level, lower baseline HBeAg level, lower HBsAg at TW12, and higher baseline ALT were associated with HBeAg seroconversion at FW24. However, these predictors had no association with the HBeAg seroconversion rate at FW24 in multivariate analysis. Similar results were also observed in the PP population.

**Table 4:** Univariate and multivariate analyses of factors associated with HBeAg seroconversion at FW24 (ITT population)

	Univariate		Multivariate^g^	
	OR	95% CI	OR	95% CI
Treatment group (1 vs. 3) Treatment group (2 vs. 3)	1.778 4.571	0.134, 23.517 0.409, 51.132	0.657 0.018	0.005, 90.457 < 0.001, 117.172
Gender^a^	1.250	0.197, 7.921		
Age (years)	1.064	0.954, 1.187		
Baseline Weight (kg)	0.993	0.940, 1.049		
Baseline HBV DNA level (log_10_ IU/mL)	0.118	0.017, 0.803	15.858	0.039, > 999.999
Baseline HBV DNA level (log_10_ copies/mL)^b^	3.599	0.371, 34.924		
Baseline HBeAg level in log_10_ scale	0.065	0.008, 0.542	0.002	< 0.001, 7.155
Baseline HBsAg^d^	6.000	0.624, 57.681		
HBsAg at TW12^e^	16.500	1.353, 201.288	121.313	0.167, > 999.999
HBsAg at TW24^e^	5.000	0.728, 34.345		
Baseline ALT^c^	9.333	1.362, 63.953	8.299	0.360, 191.193
Any ALT increase during treatment > two-fold of baseline^f^	0.278	0.029, 2.696		

*OR* odds ratio

^a^Man vs. woman

^b^ < 9 vs. ≥ 9 Log copies/mL

^c^ ≥ 5 × ULN vs. < 5 × ULN

^d^ ≤ 20,000 vs. > 20,000 IU/mL

^e^ < 1500 vs. ≥ 1500 IU/mL

^f^Yes vs. No

^g^The coefficient for the intercept is − 8.141

## Discussion

Patients treated with ropeginterferon alfa-2b 450 μg achieved the earliest time to HBeAg seroconversion (starting from TW16), followed by those treated with ropeginterferon alfa-2b 350 μg (starting from TW24). Patients treated with PEG-IFN alfa-2a 180 μg had the latest time to HBeAg seroconversion (starting from TW48). This might be explained by the higher dose of interferon in the ropeginterferon alfa-2b groups, which is supported by the finding of Liaw et al, that the patients receiving PEG-IFN alfa-2a 180 μg achieved HBeAg seroconversion earlier than those receiving 90 μg [16].

The rates of HBeAg seroconversion at follow-up were 36.4% (4/11) in ropeginterferon alfa-2b 450 μg, followed by 27.3% (3/11) in ropeginterferon alfa-2b 350 μg, and 11.1% (1/9) in PEG-IFN alfa-2a 180 μg. The HBeAg seroconversion rate in patients treated with PEG-IFN alfa-2a 180 μg in this study was lower than rates reported in the previous studies (11.1% vs. 32–36.2%) [5, 16]. Compared to the registration trial of PEG-IFN alfa-2a, one possible reason is the mean age of patient in this study is approximately 11 years older than mean age of patients in the previous studies (mean aged 43–45 vs. 32–34 years). This implies that HBeAg seroconversion rate might be even higher in the ropeginterferon alfa-2b groups if the patient population is younger.

In the registration trial of PEG-IFN alfa-2a, around 15% of HBeAg positive patients in PEG-IFN alfa-2a plus placebo group have HBeAg seroconversion at treatment week 24 (TW24) and 27% at TW48 [5]. In our study, in ropeginterferon alfa-2b groups (350 μg and 450 μg), 18.2% and 27.3%, respectively, had HBeAg seroconversion at TW24, 18.2% and 36.4%, respectively, at TW48. Thus, earlier HBeAg seroconversion was observed in ropeginterferon alfa-2b groups at most of the time-point when compared with PEG-IFN alfa-2a of the PEG-IFN alfa-2a registration trial. However, this possibly an earlier HBeAg seroconversion by ropeginterferon alfa-2b deserves further validation in the future trials. Nevertheless, HBeAg seroconversion among HBeAg-positive chronic HBV patients is an important treatment endpoint because it is associated with better long-term outcomes, including higher rate of HBsAg seroclearance, durable clinical remission, and slower rates of progression of liver diseases.

Our study showed that lower baseline HBV DNA level, lower baseline HBeAg level, higher baseline ALT, and lower HBsAg at TW12 were associated with HBeAg seroconversion in univariate analysis. However, these factors had no association with HBeAg seroconversion in multivariate analysis, which might be due to the small sample size of this study. Nevertheless, combination of immune-related parameters, e.g. toll-like receptors or anti-HBc antibody titer, might serve as better predictors for the response to PEG-IFN therapy [17–19]. Among the HBeAg-positive patients with HBeAg seroconversion, we observed relationships of mean increased ALT followed by decline of mean HBV DNA, indicating immunological responses to clear the virus.

The finding of the numerically lower incidence of rash in both the groups of ropeginterferon alfa-2b (9.5% and 4.5%, respectively) as compared to PEG-IFN alfa-2a (36.8%) might be explained by either the new formulation or by a more homogenous pegylated interferon isoforms. During the study period, only one serious adverse event (SAE) was observed in ropeginterferon alfa-2b 350 μg treatment group. The patient was hospitalized due to myocardial infarction and was treated by percutaneous intervention. He had the risk factors of smoking and hyperlipidemia. Overall, the majority of TEAEs reported in ropeginterferon alfa-2b groups were observed in PEG-IFN alfa-2a group. In addition, no new or unexpected TEAE was reported for ropeginterferon alfa-2b. One patient in Group 1 experienced acute flare [ALT 591 U/L and AST 252 U/L, with HBV DNA 421,000,000 IU/mL, and HBsAg 38,050.61 IU/mL] at FW12. The investigator decided to prescribe Telbivudine at his discretion, as the patient had discontinued the interferon therapy for 12 weeks.

IFN-α has immunomodulatory and has been used to treat chronic HBV since 1976 [20]. Modification of IFN through the addition of polyethylene glycol molecule leads to improved pharmacokinetic and pharmacodynamic properties and has largely replaced conventional IFN. In 2003, Cooksley et al. [21] for the first time demonstrated PEG-IFN alfa-2a (40 kDa) had improved efficacy over conventional interferon in treating chronic HBV. Albinterferon alfa‐2b is an 85.7 kDa protein consisting of recombinant human IFN alfa‐2b genetically fused to recombinant human albumin and can be administrated every two to four weeks. Although studies had shown the non-inferiority of albinterferon alfa-2b for chronic hepatitis C as compared to PEG-IFN alfa, its use on-market was prohibited by lung toxicities, e.g. fibrosing alveolitis, hemoptysis, bronchospasm or interstitial lung disease [9, 10]. By contrast, ropeginterferon alfa-2b did not show the above adverse events of lung after being exposed to 102 healthy subjects, 214 patients with polycythemia vera (including 127 patients in a phase III study in which 95 of them continuously been treated for 36 months) [22], and 270 patients with chronic viral hepatitis (unpublished data). The safety profile is therefore validated. Furthermore, in the phase III study [22], the drug was well tolerated in an average of 48–67 years old PV patients. Similar benefits are expected in hepatitis B patients, a population growing older worldwide.

There are several limitations of this study. Firstly, the small sample size of this study on ropeginterferon alfa-2b in chronic HBV patients. Small sample size may reduce sensitivity of the analysis, e.g. the effect of ropeginterferon alfa-2b on HBsAg loss; and introduce some attrition bias. Secondly, HBV genotype was not tested in this study. Although HBV genotype had been reported to correlate with clinical outcomes and response to PEG-IFN therapy [23], it has not been widely used clinically. Furthermore, studies had confirmed chronic HBV patients in Taiwan were infected by genotype B and C [24].

The enthusiasm for interferon has recently been revived in many recent clinical trials by combining small molecules with interferon for hepatitis B or D to maximize treatment responses [25]. Interferon would be the backbone of treatment in patients with chronic hepatitis D. Although most of patients with CHB are under NAs treatment, the unmet medical need is that the rate of HBsAg loss is low. Recent many new clinical trials for hepatitis B combine small molecules or other agents with interferon to maximize the therapeutic efficacy [26–30]. We anticipate a renewed interest in interferon as safe and reliable immunomodulator therapy in combination with emerging anti-HBV regimens.

Among the two dose levels of ropeginterferon alfa-2b, the 450 µg had higher cumulative HBeAg seroconversion rate of 36.4% at follow-up period (versus 27.3% in 350 µg), with the time to HBeAg seroconversion earlier at treatment week (TW) 16 in the 450 µg group (versus TW24 in the 350 µg group). In safety, the side effects among the two ropeginterferon alfa-2b groups were comparable, including the rash. However, the rate of ALT elevation was only higher in ropeginterferon alfa-2b 350 μg group (38.1%), while the rate of ALT elevation was similar in ropeginterferon alfa-2b 450 μg and PEG-IFN alfa-2a 180 μg (9.1% versus 10.5%). Due to small case number in this study, final dose selection will need further clinical trials to decide.

In conclusion, this preliminary study revealed that ropeginterferon alfa-2b, although in only half the number of injections, is tolerable with comparable safety and efficacy to PEG-IFN alfa-2a. The results show that patients’ time and visits can be saved, and lays the groundwork in developing this new regimen of interferon-based therapy for chronic hepatitis B or hepatitis D.

## Electronic supplementary material

Below is the link to the electronic supplementary material.

Supplementary file1 (DOCX 24 kb)
