# Supplementary material for: Ropeginterferon alfa-2b every 2 weeks as a novel pegylated interferon for patients with chronic hepatitis B
Source: Hepatol Int. 2020 Oct 24;14(6):997–1008. doi: 10.1007/s12072-020-10098-y (PMC7803873; doi:10.1007/s12072-020-10098-y)
Supplement: Supplementary file 1 — Supplementary file1 (DOCX 24 kb) [file 12072_2020_10098_MOESM1_ESM.docx]

**Supplemental Table 1.** Patient Number & Reason of Screen Failure

| **Not eligible for  Inclusion Criteria** | **Reason** | **Patient Number** |
| --- | --- | --- |
| #2 ^a^ | ALT level did not meet the criteria | 38 |
| #2 ^a^ | HBV DNA level did not meet the criteria | 13 |
| #3 ^b^ | Total bilirubin level did not meet the criteria | 2 |
| #6 ^c^ | Positive for anti-HDV | 1 |
| #7 ^d^ | Fundoscopic examination did not meet the criteria | 2 |
| **Screen out by  Exclusion Criteria** | **Reason** | **Patient Number** |
| #3 ^e^ | Clinically significant abnormal laboratory test | 1 |
| #3 ^e^ | Clinically significant abnormal WBC level | 1 |
| #10 ^f^ | Poorly controlled diabetes | 1 |
| **Others** | **Reason** | **Patient Number** |
| Withdraw consent | N/A | 2 |

^a^: Inclusion criteria #2: Confirmed diagnosis of chronic hepatitis B (CHB) virus infection: (1) Positive for HBsAg for at least 6 months, and (2) documented elevated ALT, i.e. 1.5-10 X ULN, and (3) serum HBV DNA >20,000 IU/mL for both HBeAg(+) and HBV DNA >2,000 IU/mL for HBeAg(-) at screening visit.

^b^: Inclusion criteria #3: Compensated liver disease, which includes but not limited to the following: total bilirubin<2 mg/dl (except in Gilbert syndrome), albumin level (≥ 3.5 g/dL), normal albumin, normal INR (INR ≤1.5); no clinical evidence of ascites, cirrhosis, liver decompensation,hepatic encephalopathy, esophageal varices or portal hypertension as identified by ultrasound or any other procedures before study entry.

^c^: Negative for human immunodeficiency virus, hepatitis C and hepatitis D infection.

^d^: Normal fundoscopic examination at screening, defined as no significant or major fundoscopic findings including but not limited to retinal exudates, hemorrhage, detachment, neovascularization, papilloedema, optic atrophy, microaneurysms and macular changes.

^e^: Clinically significant abnormal laboratory test result at screening which includes but is not limited to the followings: WBC < 3,000/mm^3^, ANC < 1500/mm^3^, Hgb < 10g/dL, platelet <90,000/mm^3^ or other abnormal values that should be excluded at the investigator’s discretion.

f: Any presence of poorly controlled major psychiatric (including but not limited to those with severe depression, severe bipolar disorder, schizophrenia, suicidal ideation or history of suicidal attempt), neurological, cardiovascular (e.g. uncontrolled hypertension), pulmonary (including but not limited to chronic obstructive lung disease), hematological, immunologic, endocrine, metabolic or other uncontrolled systemic disease, coagulation disorders or blood dyscrasias.

N/A: Not Applicable

Supplemental Table 2. Efficacy Summary (PP population)

|  | **HBeAg (+)** | | | | | | | **HBeAg (-)** | | | |
| --- | --- | --- | --- | --- | --- | --- | --- | --- | --- | --- | --- |
|  | **P1101/350 ug**  **(N=7)** | | **P1101/450 ug**  **(N=10)** | | **Peg-IFN alfa-2a**  **180 ug (N=8)** | |  | **P1101/350 ug**  **(N=9)** | **P1101/450 ug**  **(N=11)** | **Peg-IFN alfa-2a**  **180 ug (N=8)** |  |
| **HBV DNA Level  < 2,000 IU/mL** | |  | |  | |  |  |  |  |  |  |
| **TW4** | | **0 (0.0)** | | **0 (0.0)** | | **0 (0.0)** |  | **8 (88.9)** | **6 (54.5)** | **5 (62.5)** |  |
| **TW8** | | **1 (14.3)** | | **0 (0.0)** | | **0 (0.0)** |  | **8(88.9)** | **7 (63.6)** | **8 (100.0)** |  |
| **TW12** | | **1 (14.3)** | | **1 (10.0)** | | **1 (12.5)** |  | **9 (100.0)** | **9 (81.8)** | **8 (100.0)** |  |
| **TW24** | | **2 (28.6)** | | **1 (10.0)** | | **1 (12.5)** |  | **9 (100.0)** | **9 (81.8)** | **8 (100.0)** |  |
| **TW48** | | **3 (42.9)** | | **4 (40.0)** | | **2 (25.0)** |  | **9 (100.0)** | **10 (90.9)** | **8 (100.0)** |  |
| **FW12** | | **2 (28.6)** | | **1 (10.0)** | | **1 (12.5)** |  | **3 (33.3)** | **6 (54.5)** | **5 (62.5)** |  |
| **FW24** | | **2 (28.6)** | | **0 (0.0)** | | **2 (25.0)** |  | **1 (11.1)** | **4 (36.4)** | **5 (62.5)** |  |
| **HBsAg Levels  < 1,500 IU/mL** | |  | |  | |  |  |  |  |  |  |
| **TW12** | | **1 (14.3)** | | **2 (20.0)** | | **1 (12.5)** |  | **4 (44.6)** | **7 (63.6)** | **8 (100.0)** |  |
| **TW24** | | **2 (28.6)** | | **2 (20.0)** | | **2 (25.0)** |  | **6 (66.7)** | **8 (72.7)** | **8 (100.0)** |  |
| **TW48** | | **4 (57.1)** | | **3 (30.0)** | | **3 (37.5)** |  | **8 (88.9)** | **9 (81.8)** | **8 (100.0)** |  |
| **FW24** | | **4 (57.1)** | | **2 (20.0)** | | **1 (12.5)** |  | **8 (88.9)** | **10 (90.9)** | **8 (100.0)** |  |
| **HBeAg Seroconversion** | |  | |  | |  |  |  |  |  |  |
| **TW24** | | **1 (14.3)** | | **3 (30.0)** | | **0 (0.0)** |  | **NA** | **NA** | **NA** |  |
| **TW48** | | **1 (14.3)** | | **4 (40.0)** | | **1 (12.5)** |  | **NA** | **NA** | **NA** |  |
| **FW12** | | **2 (28.6)** | | **4 (40.0)** | | **1 (12.5)** |  | **NA** | **NA** | **NA** |  |
| **FW24** | | **2 (28.6)** | | **4 (40.0)** | | **1 (12.5)** |  | **NA** | **NA** | **NA** |  |

**NA = Not applicable; P1101 = Ropeginterferon alfa-2b.**

|  | **Univariate** | | **Multivariate^$^** | |
| --- | --- | --- | --- | --- |
|  | **OR** | **95% CI** | **OR** | **95% CI** |
| **Treatment Group (1 v. 3)**  **Treatment Group (2 v. 3)** | **2.800**  **4.666** | **0.196, 40.049**  **0.404, 53.939** | **42.621**  **<0.001** | **<0.001, >999.999**  **<0.001, >999.999** |
| **Gender^#^** | **1.250** | **0.185, 8.444** |  |  |
| **Age (years)** | **1.050** | **0.940, 1.171** |  |  |
| **Baseline Weight (kg)** | **1.006** | **0.945, 1.072** |  |  |
| **Baseline HBV DNA level (log_10_ IU/mL)** | **0.133** | **0.019, 0.946** | **>999.999** | **<0.001, >999.999** |
| **Baseline HBV DNA level (log_10_ copies/mL)*** | **3.818** | **0.375, 38.822** |  |  |
| **Baseline HBeAg level in log_10_ scale** | **0.078** | **0.009, 0.653** | **<0.001** | **<0.001, >999.999** |
| **Baseline HBsAg^##^** | **6.000** | **0.596, 60.439** |  |  |
| **HBsAg at TW12^@^** | **12.748** | **1.034, 157.107** | **>999.999** | **<0.001, >999.999** |
| **HBsAg at TW24^@^** | **3.750** | **0.537, 26.188** |  |  |
| **Baseline ALT**** | **10.666** | **1.309, 86.927** | **>999.999** | **<0.001, >999.999** |
| **Any ALT increase during treatment > 2 fold of baseline^&^** | **0.333** | **0.032, 3.436** |  |  |

Supplemental Table 3. Univariate and Multivariate Analyses of Factors Associated With HBeAg seroconversion at FW24 (PP population)

**OR = Odds ratio; P-value: Wald test; ^#^: man vs. woman; *: <9 vs. ≥9 Log copies/mL; **:≥5×ULN vs. <5×ULN; ##: ≤20,000 vs. >20,000 IU/mL; @:<1500 vs. ≥1500 IU/mL; &: Yes vs. No;**

**$: The coefficient for the intercept is -50.187.**
